# Supplementary material for: Patterns of Intron Gain and Loss in Fungi
Source: PLoS Biol. 2004 Nov 30;2(12):e422. doi: 10.1371/journal.pbio.0020422 (PMC532390; doi:10.1371/journal.pbio.0020422)
Supplement: Table S1 — Also available at http://genes.mit.edu/NielsenEtAl/. (4.3 MB ZIP). [file pbio.0020422.st001.zip › NielsenEtAl/html/223.html]

AN9459.1.NCU01381.1.MG09526.1.FG06088.1


```
 CLUSTAL W (1.82) Multiple Sequence Alignments - Introns Inserted


Sequence 1: NCU01381.1	186 aa
Sequence 2: FG06088.1	111 aa
Sequence 3: MG09526.1	177 aa
Sequence 4: AN9459.1	211 aa
Alignment Length: 268 aa
Number Identitical Residues: 35 aa
Alignment Score (without introns) 2186


MG09526.1 	----------------------~-----MASEVFFTVCFRMANPPQALKSEYTFRPAILH
NCU01381.1	MANPEQPSAQSDDPVHRAFFYA1RKGTLMVPEVFFTVCYNLKIVPDVVARQHVFTPAILH
FG06088.1 	----------------------~-----------------------------------MQ
AN9459.1  	----------------------~-----MAPQILHRVIHGSPTPQQWQKDLLQFRPAVLH
          	                              ..             .         .: ::

MG09526.1 	~GYCRRRVK2DADYPGIVPDAE----------------------------HTVRGTYVTG
NCU01381.1	~GYCRRRVK~SADYPGITEDAG----------------------------HSVFGMLAEG
FG06088.1 	1GYCRHRVR~RADYPGVVAEKG----------------------------HNVRGVYATG
AN9459.1  	~GYRRHRVR~GADYPGIIRESESTQDAATDGSESATGKAALPSGPETRSRASVLGTVVSG
          	 ** *:**:  *****:  :  ::..:::..:.:::..:: .:...: :  .* *  . *

MG09526.1 	LTEANLVKLDFFEGSEYDRLPVKVKLL---------EKVGDDKGEGN-------------
NCU01381.1	LTKANLDKLDIFEGAEYERRTVKVKVL---------KKVGDVTGEGN-------------
FG06088.1 	LTDANMSKLDDFEGPEYDKEKVKVKLL---------NKDGTVNEKG--------------
AN9459.1  	LTDGDIHRLDIFEGAEYRREKVSVRILREALSKDLNNQDGVRNGDENRAHVDADAARDKH
          	**..:: :** ***.** :  *.*::* .: :.. .:: *  . . . :  .:.:: .. 

MG09526.1 	-------------VEGAEVETSTYVFKD-KALLEDKEWDFDHFRNERMKHWTREDYC~FS
NCU01381.1	-------------MEGEEKDAQVYVFLP-TQHLEQKEWDLEEFKREKLKLWTRGDHI~FA
FG06088.1 	----------------ETKETTAYVFNS-LNHLEKREWDFEEFRKTKMKNWTRGGLA~FD
AN9459.1  	IKDVLEAAGADFADEEEEVETETYVWIAGRTMLEDAEWDFEAFKRDKMAWWVSADEK1CV
          	 ..  .::.:. : .    :: .**:  .   **. ***:: *:. ::  *.  .     

MG09526.1 	~G1GEPVDARGKHGLHDQPWRTEGMQRAVASGSS
NCU01381.1	~D1CDPEEPA-----------------TVAAAV-
FG06088.1 	~-~-------------------------------
AN9459.1  	0S~F------------------------------
          	 .
```
